# Development of a Lexicon for Pain

**DOI:** 10.3389/fdgth.2021.778305

**Published:** 2021-12-13

**Authors:** Jaya Chaturvedi, Aurelie Mascio, Sumithra U. Velupillai, Angus Roberts

**Affiliations:** ^1^Department of Biostatistics and Health Informatics, Institute of Psychiatry, Psychology and Neurosciences, King's College London, London, United Kingdom; ^2^Health Data Research UK, London, United Kingdom

**Keywords:** lexicon, natural language processing, pain, electronic health records, mental health

## Abstract

Pain has been an area of growing interest in the past decade and is known to be associated with mental health issues. Due to the ambiguous nature of how pain is described in text, it presents a unique natural language processing (NLP) challenge. Understanding how pain is described in text and utilizing this knowledge to improve NLP tasks would be of substantial clinical importance. Not much work has previously been done in this space. For this reason, and in order to develop an English lexicon for use in NLP applications, an exploration of pain concepts within free text was conducted. The exploratory text sources included two hospital databases, a social media platform (Twitter), and an online community (Reddit). This exploration helped select appropriate sources and inform the construction of a pain lexicon. The terms within the final lexicon were derived from three sources—literature, ontologies, and word embedding models. This lexicon was validated by two clinicians as well as compared to an existing 26-term pain sub-ontology and MeSH (Medical Subject Headings) terms. The final validated lexicon consists of 382 terms and will be used in downstream NLP tasks by helping select appropriate pain-related documents from electronic health record (EHR) databases, as well as pre-annotating these words to help in development of an NLP application for classification of mentions of pain within the documents. The lexicon and the code used to generate the embedding models have been made publicly available.

## Introduction

**Figure 1:**
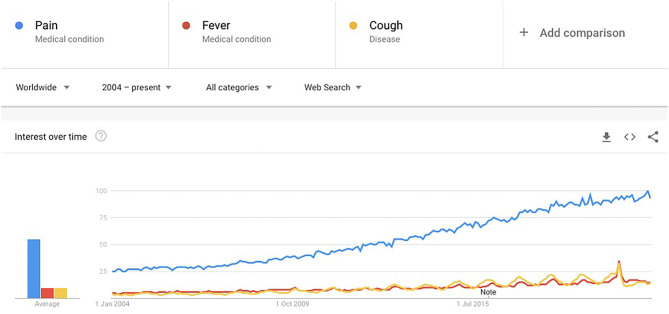
Google trends for medical condition search term “pain” compared to other common symptoms “fever” and “cough.” X-axis represents time in years. Y-axis numbers represent the search interest relative to the highest point on the chart (100 is the peak popularity for the term, 50 indicates the term is half as popular, and 0 means there was insufficient data for the term).

**Figure 2:**
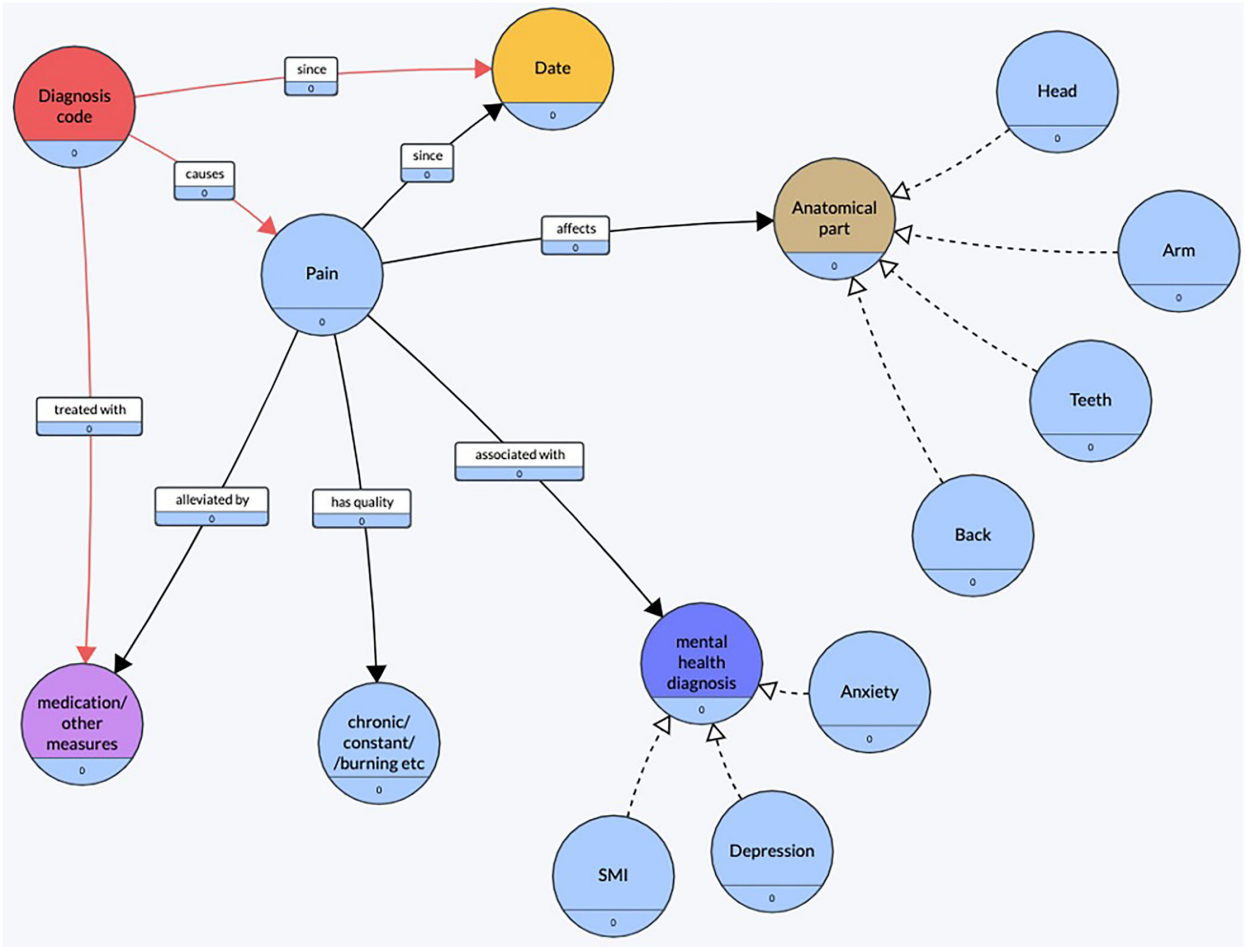
Conceptual diagram of pain. Created using an online tool, Grafo (43).

Pain is known to have a strong relationship with emotions, which can lead to damaging consequences (1). This is worsened for people suffering with persistent pain. It can lead to long-term mental health effects such as “secondary pain effect” which encapsulates the strong feelings toward the long-term implications of suffering from pain (1). The Biopsychosocial framework of pain reiterates the multidimensionality of pain and explains the dynamic relationships of pain with biological, psychological, and social factors (2). Pain has been an active area of research, especially since the onset of the crisis of opioid use in the United States (3). Pain also has a significant impact on the healthcare system and society in terms of costs (4). Apart from research, it has also been of interest to the general population. **Figure 2** shows Google trends for the search term “pain” over time (2004 to present) compared with two other common symptoms (“fever” and “cough”) to investigate whether the trends are reflective of a general increase in searches, or an actual increase in search of the term. All three terms were selected as “medical terms” rather than “general search” terms to avoid any metaphorical mentions and make the words more accurately comparable. This was possible through use of a Google Trends feature which allows the user to choose the search category (generic “Search term” category would include any search results for the word “pain,” “Medical condition”/“Disease” category would only include “pain” when searched as a medical condition or disease). Pain shows an incremental increase worldwide (Figure 1) (5).

Research is a growing secondary use of mental health electronic health records (EHRs), specifically the free-text fields (6). It has the potential to provide additional information on contextual factors around the patient (7). While it is beneficial to include clinical notes in research, extracting, and understanding information from the free text can be challenging (8). Natural language processing (NLP) methods can help combat some of the issues inherent in clinical text, such as misspellings, abbreviations, and semantic ambiguities.

Another rich source of health-related textual data is social media as it provides a unique patient perspective into health (9). In recent years, there has been an increase in the use of social media platforms to share health information, receive and provide support, and look for advice from others suffering with similar ailments (9). Content from these platforms has also been increasingly used in health research. Examples include finding symptom clusters for breast cancer (10), understanding the relationships between e-cigarettes and mental illness (11), as well as understanding user generated discourse around obesity (12). The main platforms involved in these studies have been Reddit^1^ and Twitter^2^ Reddit has been a good source for such textual research due to its wide usage as well as the ability to post anonymously (13). Reddit has more than 330 million active monthly users, and over 138K active communities (14). A key feature of Reddit is the subforum function which allows creation of subreddit communities dedicated to shared interests (9). Twitter has shorter text spans than Reddit, a maximum of 280 characters (15). Despite this limitation, Twitter is widely used in research around mental health and suicidality (16–18).

The term “pain” presents a unique NLP problem, due to its subjective nature and ambiguous description. Pain can refer to physical distress, or existential suffering, and sometimes even legal punishment (19). However, within the clinical context, it will most likely be the former two. It also has metaphorical uses in phrases such as “for being a pain” (19). In order to better understand how pain is described in different textual sources, and to construct a lexicon of pain for use in NLP applications, this study does a preliminary exploration of mentions of pain. This exploration includes analysis of mentions of pain in four different sources with the objective of understanding how mentions of pain differ in these sources, and whether they cover common themes. These exploratory sources include—a mental health hospital in the UK (CRIS, from the South London and Maudsley NHS Foundation Trust), the critical care units of a hospital in the United States (MIMIC-III), Reddit, and Twitter.

Gaining a good understanding of how pain is mentioned in text can be formalized by creation of a lexicon of pain terms. Lexicons are a valuable resource that can help develop NLP systems and improve extraction of concepts of interest from clinical text (20). Lexicons provide a wide range of terms and misspellings from relevant domains, which will be advantageous in future NLP tasks and will minimize the risk of missing important documents that contain these relevant terms. An existing ontology, The Experimental Factor Ontology (21), consists of a subsection of 26 pain related terms, but to our knowledge, no previous studies have explored how the concept of pain is used in different text sources, and used this to generate a new lexicon. While using terms generated by a domain expert has the benefit of being more precise, we believe that for an ambiguous term such as pain, our method of producing a lexicon semi-automatically, for domain expert review, will favor recall without damaging precision (i.e., sensitivity without loss of positive predictive value). The generation of this lexicon will involve a combination of terms related to pain from three sources—literature, ontologies, and embedding models built using EHR data. Mentions from social media that were part of the exploratory sources are not included as lexicon sources since the primary purpose of the lexicon in this instance is for use on EHR data. Any relevant mentions from social media may be added to the lexicon at a later date.

The aim of this study was to conduct an exploration of how pain was mentioned within four different text sources. The purpose of this exploration was to understand what sources of textual information might be useful additions to the lexicon. The eventual goal of generating this lexicon is to be able to use it in downstream NLP tasks where it can be used to identify relevant pain-related documents from EHR databases.

## Materials and Methods

The final lexicon consists of relevant pain related terms from three key areas—ontologies, literature, and embedding models. The lexicon was reviewed and validated by domain experts. In addition to this, the lexicon was also compared to another ontology that consists of 26 pain-related terms. This ontology is available as part of the Experimental Factor Ontology (version 1.4) (21) as a subsection for pain.

### Data Collection and Exploration/Source Comparison

Four different data sources were explored for mentions of pain within their textual components, and a comparison was conducted to understand the different contexts in which pain can be mentioned. Fifty randomly selected documents were extracted from each source. The number of documents was limited to 50 per text source for pragmatic reasons: manual review is a labor-intensive process. This decision should not impact the lexicon development, as these documents are used only for exploration, with embeddings built on the whole of two sources (MIMIC and CRIS) were used to generate the terms for the lexicon to supplement the development of the lexicon.

#### Ethics and Data Access

While data from Reddit and Twitter are publicly available, applicable ethical research protocols proposed by Benton et al. were followed in this study (22). No identifiable user data or private accounts were used, and any sensitive direct quotes were paraphrased.

Data from Twitter is available through their API after approval of registration for access to this data, details of which can be found in their general guidelines and policies documentation (23). Data access information for CRIS (24) and MIMIC-III (25) are detailed on their respective websites.

#### CRIS

An anonymized version of EHR data from The South London and Maudsley NHS Foundation Trust (SLaM) is stored in the Clinical Record Interactive Search (CRIS) database (6). The infrastructure of CRIS has been described in detail (26) with an overview of the cohort profile. This project was approved by the CRIS oversight committee (Oxford C Research Ethics Committee, reference 18/SC/0372). Clinical Record Interactive Search consists of almost 30 million notes and correspondence letters, with an average of 90 documents per patient (7).

A SQL query was run on the most common source of clinical text (“attachments” table which consists of documents such as discharge and assessment documents, GP letters, review, and referral forms) within the CRIS database, and 50 randomly selected documents that contained the keyword “pain” (both upper and lower case) were extracted. This would include any instance of “pain” regardless of whether it refers to physical pain or emotional/mental pain. Other features of the documents, such as maximum and minimum length of documents were calculated, as well as common collocates for the term “pain.”

#### MIMIC-III

Medical Information Mart for Intensive Care (MIMIC-III) is an EHR database which was developed by the Massachusetts Institute of Technology (MIT), available for researchers under a specified governance model (25). Medical Information Mart for Intensive Care consists of about 1.2 million clinical notes (27).

A SQL query was run on the “note-events” table which contains majority of the clinical notes (such as nursing and physician notes, ECG reports, radiology reports, and discharge summaries) within the database, and 50 random documents containing the keyword “pain” (both upper and lower case) were extracted. Like the CRIS database, an analysis of the maximum and minimum length of documents was carried out, and common collocates for the term “pain” were explored.

#### Reddit

Reddit is an online community which supports unidentifiable accounts to allow users to post anonymously and provides sub communities for people to discuss topics of shared interest. The chronic pain subreddit (r/ChronicPain) community was used in this study. Other subreddits around pain included more specific communities, such as “back pain,” which would not serve our purpose of keeping it general. While this approach might miss mentions of other types of pain, there didn't seem to be a way around this due to absence of a general pain subreddit. Data from Reddit was extracted using the python package PRAW (28). No time filter was applied. Seven thousand seven hundred posts were extracted, out of which 50 posts were randomly selected.

#### Twitter

Twitter is an online micro-blogging platform with an enormous number of users who post short (280 characters or less) messages, referred to as “tweets,” on topics of interest. It is a good resource for textual data because of the volume of tweets posted on it and the public availability of this data (29). Python package tweepy (30) was used to extract tweets using the search term “chronic pain.” As with Reddit, chronic pain was used instead of pain to help get more meaningful health-related results. This approach was not applied to the EHR text as the assumption was that metaphorical mentions would be more prevalent in social media. This does carry the risk of possibly missing out on mentions of pain that were not explicitly chronic. Since the Twitter API allows for extraction of tweets within a seven day window, 7,707 tweets were extracted within the time period 06/08/2020 to 11/08/2020 that consisted of the keywords “chronic pain” (case insensitive). Out of these, 50 tweets were randomly selected for analysis.

### Lexicon Development

Concordances and analyses on data from the previous step were used to inform the appropriateness of the mentions of “pain” and whether they were meaningful mentions and thereby suitable for inclusion in building a lexicon of pain terms. The terms within the EHR text had more appropriate concordances (i.e., referring to actual pain rather than metaphorical mentions) and were therefore included in the lexicon while the social media ones were not. Embedding models built using Twitter (31) and Reddit (32) data were not used as their results returned words that did not seem relevant to the term “pain.” They generated terms such as brain, anger, patience, and habit with Twitter, and words such as apartment, principal, and goal by Reddit. In addition to this, a few publications and ontologies were explored as potential sources as well. The final lexicon was built by combining terms generated through three different sources.

#### Literature-Based Terms

We harvested pain-related words from three publications:

(1) A list of symptom terms provided by a systematic review on application of NLP methods for symptom extraction from electronic patient-authored text (ePAT) (33). Some examples include pain, ache, sore, tenderness, head discomfort.
(2) Ten words most similar to pain generated in a survey of biomedical literature-based word embedding models (34). Some examples include discomfort, fatigue, pains, headache, backache.
(3) A list of sign and symptom strings generated using NLP to meaningfully depict experiences of pain in patients with metastatic prostate cancer, as well as identify novel pain phenotypes (1). In our literature search, this was the only paper on NLP-based extraction of pain terms that included a list of the terms used. Some examples include ache, abdomen pain, backpain, arthralgia, bellyache.

These lists were cleaned by lowercasing all terms, and only keeping terms made up of one or two tokens as these included most of the terms, and any terms with more than two tokens were less meaningful or repetitive of the two token terms. Terms with more than two tokens were only listed in one of the papers (1), and some examples of these were terms such as pain of jaw, right lower quadrant abdominal pain, upper chest pain, and so on, most of which were covered within the two token terms such as abdominal pain and chest pain.

#### Ontology-Based Terms

We incorporated synonyms for pain from three biomedical ontologies—The Unified Medical Language System (UMLS) (35), Systematized NOmenclature of MEDicine Clinical Terms (SNOMED-CT) (36), and International statistical Classification of Diseases and related health problems: tenth revision (ICD-10) (37). Unified Medical Language System contains concepts from SNOMED-CT and ICD-10, in addition to several other vocabularies. From each, we extracted terms of up to two tokens that either matched “pain^*^,” were synonyms of pain, or described as child nodes of pain.

#### Embedding Models

Embedding models (38, 39) using eight different parameters and four different text sources were used to generate additional words similar to “pain.” The elbow method (40) was used to determine the cut-off point in word similarity which helped determine the similarity threshold for each model. An advantage of using embedding models is their ability to capture misspellings. Any duplicates were removed, and the remaining terms were added to the lexicon.

Two of the embedding models [both described in Viani et al. (41)] were built using clinical text available within the MIMIC-II database (42). Four embedding models were built using clinical text available within MIMIC-III, of which three were built using genism implementation of word2vec (38) and one using FastText (43). One model was built using word2vec over a severe mental illness (SMI) cohort from CRIS. Finally, a publicly available model built on PubMed and PubMed Central (PMC) article texts was used (44). Only unigrams were included from all the models. The parameters for these are detailed in **Table 6**.

### Validation

Upon collection of data from the four different sources, common themes were explored. The purpose was to understand the common contexts in which pain might be mentioned. In addition to common themes, length of the text containing mentions of pain was calculated, along with most frequent concordances and mutual information scores.

Validation of the terms for inclusion in the final lexicon was conducted using two methods—validation by two clinicians, comparison to an existing pain-related lexicon, and comparison to MeSH (Medical Subject Headings)^3^.

A list of the terms generated through the three text sources was shared with two clinicians who marked each term as: relevant mention of pain, not relevant to pain, or too vague in relation to pain. In addition to this, they added a few new terms to the lexicon.

As an additional validation step, the final lexicon validated by the clinicians was compared to an existing ontology, The Experimental Factor Ontology (21), which consists of a sub-section of 26 pain-related terms. The final lexicon was also compared to 63 pain-related MeSH terms. Each MeSH term also consisted of a set of entry terms (a total of 941 pain-related terms). Entry terms refer to synonyms, alternate forms, and other terms that are closely related to the MeSH term (45). With both these comparisons, any terms that did not overlap were investigated to see why they might be missing from our lexicon and any terms that did not overlap were investigated to see why they might be missing from our lexicon.

After generation and validation of the final lexicon, the pain-related terms were separated out from the terms (such as pain from leg pain, arm pain; sore from sore mouth, sore muscle, etc.) and these terms were looked up within a cohort of SMI patients from the CRIS database. A frequency count was conducted to see which of these terms occur most frequently within this cohort of patients.

## Results

### Exploration of Pain

Three common pain terms were chosen to gain an understanding of how frequently they are mentioned in EHR documents. These terms were: pain, chronic pain, and words ending with -algia, a common suffix meaning pain. A more detailed search on other pain-related terms such as ache will be conducted at a later stage. A summary of frequencies of these terms within the two EHR-based sources is outlined in Table 1. As seen in the table, the term “pain” had the greatest number of mentions and was thus used for selecting documents from the databases for exploration (as described in the Materials and Methods section).

**Table 1:** Count of mentions of “pain”, “chronic pain,” and “-algia” per 10,000 tokens (counts for “pain” include “chronic pain” instances too).

**Terms**	**CRIS—Attachments**	**MIMIC-III**
Pain	29.59	44.13
Chronic pain	1.22	4.04
*Algia	1.14	1.44

Comparing the EHR text data to those from social media platforms Twitter and Reddit, the length of text containing the word “pain” was calculated to understand how much content might be available in each source (Table 2).

**Table 2:** Length of text within documents containing the word “pain” in the 4 text sources on a random set of 50 documents for each text source.

**Source**	**CRIS**	**MIMIC**	**Twitter**	**Reddit**
Average length of text (charac.)	8,144	3,864	62	1,065
Minimum length of text (charac.)	1,155	165	11	139
Maximum length of text (charac.)	32,767	9,549	106	3,598

During the comparison of these sources, four common themes emerged, as shown in Table 3.

**Table 3:** Common themes around “pain” in the 50 randomly selected documents from the four data sources.

**Source**	**Quality/Type of pain**	**Feelings/Experiences associated with the pain**	**Medication or other measures**	**Related to body parts**
CRIS	In constant pain Ongoing pain Pain was quite severe	Overwhelmed by chronic pain problems Fear of pain Pain causing distress Struggles with chronic pain	Drugs to numb the pain Pain relief medication not controlling the pain Side effects from pain relief medication No pain relief with NSAIDs	Chronic back pain Chest pain
MIMIC-III	Severe pain atypical pain	–	PO as needed for pain Taking narcotic pain medication Managed with IV pain medication and Pain was controlled with oral analgesics	Chronic back pain Chest pain Abdominal pain Right leg pain Chronic lower back pain
Reddit	Sharp pain Widespread pain	Could be causing pain Painful trips to the kitchen In the same painful position as 3 months ago	Helped my back pain	Shoulder pain Back pain Chronic neck pain Chronic joint pain
Twitter		To live pain-free	Muscle painbuster	Joint muscle pain Back pain

An analysis was conducted using Lancsbox (46) to get the collocates associated with the term “pain,” limiting to only those words that had a frequency of more than 10. The top five collocates from the different sources are listed in Table 4. Reddit and Twitter produced mostly generic terms which were not very meaningful.

**Table 4:** Collocates for “pain” with frequency > 10.

**CRIS**	**MIMIC-III**	**Reddit**	**Twitter**
Chronic	Control	Pain	Agony
Back	Acute	About	Amazingly
Clinic	Chronic	Anyone	Achieved
Physical	Assessment	Back	American
Health	Plan	Anything	Body

The collocation tool within LancsBox looks at five words on either side of the search term “pain,” which explains why “pain” is also a collocate within the Reddit dataset since there were instances of mentions of “pain” as can be seen in these paraphrased examples— “I suffer from a condition which causes back pain and pain in legs”; “I have chronic pain. The pain is in my shoulder…” and could also be why generic words like “anyone” (instances such as “I have tried opioids for back pain. Has anyone else seen an improvement with this…”; “Has anyone used heat for pain…”) and “anything” (instances such as “the meds are not doing anything for my pain”) have been selected.

Table 5 lists out the top five collocates for “pain” with a mutual information (MI) score >6. MI score measures the amount of non-randomness present when two words occur (47) thereby giving a more accurate idea of the relationship between two words (48). It is recommended that an MI score greater than 3 be used (48) to get more meaningful results. An MI score of 5 and more was used in this instance since collocates with a lower MI score were generic and vague, including words such as “what,” “if,” and “with.” The letters in the brackets indicate whether they occurred to the right (R) or left (L) of the word “pain.” Reddit and Twitter data produced mostly generic results.

**Table 5:** Collocates for “pain” with an MI score > 6.

**CRIS**	**MIMIC-III**	**Reddit**	**Twitter**
Killers (R)	Chronic (R)	Board (R)	People (L)
Chronic (L)	Control (L)	Certified (L)	Amp (R)
Fibromyalgia (R)	Complains (L)	Suboxone (L)	Get (L)
Ongoing (R)	Incisional (L)	Chronic (L)	Medical (L)
Feet (R)	Acute (L)	Doctor (R)	Suffer (L)

Using the observations made during this preliminary exploration, a conceptual diagram (Figure 2) of pain was created. The objective of constructing this conceptual diagram was to visualize what features were commonly found around the mention of pain.

### Building the Lexicon

Table 6 summarizes the number of words obtained from the three different sources. For the embedding models, the model parameters and elbow thresholds are also included.

**Table 6:** Number of words obtained from the different sources, and parameters/elbow threshold for the embedding models.

**Source**	**Parameters**	**Elbow threshold**	**No. of unigrams**	**No. of bigrams**	**Total no. of words**
Literature	–	–	71	170	241
Ontologies			83	440	523
UMLS	–	–	11	70	81
SNOMED-CT	–	–	67	368	435
ICD-10	–	–	5	2	7
Embedding models			171	–	171
MIMIC-II	w2v, size = 100, window = 5, min_count = 15, workers = 4	0.57	33	–	33
MIMIC-II	w2v, size = 400, window = 5, min_count = 15, workers = 4	0.47	40	–	40
MIMIC-III	w2v, size = 100, window = 5, min_count = 15, workers = 4	0.66	4	–	4
MIMIC-III	w2v, size = 400, window = 5, min_count = 15, workers = 4	0.47	12	–	12
MIMIC-III	w2v, size = 300, window = 10, min_count = 5, workers = 16	0.44	26	–	26
MIMIC-III	FastText, size = 300, window = 10, min_count = 5	0.93	30	–	30
CRIS (SMI)	w2v, size = 300, window = 10, min_count = 5	0.69	16	–	16
PubMed	w2v, size = 200, window = 5	0.73	10	–	10

After compiling the words from all these sources, the total size of the lexicon was 935 words (including duplicates and 57 misspellings), with 35% of them being unigrams and 65% bigrams. The most frequently occurring words in the final lexicon were pain (*n* = 46), discomfort (*n* = 10), headache (*n* = 8), soreness (*n* = 8), and pains/painful/ache/backache (*n* = 7). Table 7 shows the coverage of the lexicon at this stage.

**Table 7:** Lexicon coverage.

**Lexicon source**	**No. of unique terms**	**Total no. of terms**
Literature	218	241
Ontologies	291	523
Embeddings	68	171

**Figure 3:**
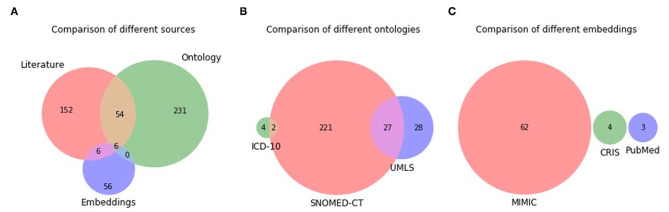
Venn diagram of unique terms generated from the different sources **(A)**, different ontologies **(B)**, and different embedding models **(C)**.

The Venn diagrams of the unique terms are shown in Figure 3. A total of six terms overlap between the three sources, with the most overlap (54 terms) being between literature and ontology. There is no overlap between all three ontologies, with the most overlap (27 terms) being between SNOMED-CT and UMLS. There is no overlap between ICD-10 and UMLS due to the former consisting of mostly three-token terms, while the terms in all sources have been limited to up to two tokens. For example, ICD-10 consists of terms such as pain in limb, pain in throat, pain in joints, rather than limb pain, throat pain, and joint pain. There was no overlap at all between the different embedding models. A comparison of the two MIMIC models (MIMIC-II and MIMIC-III) showed that they generated unique terms with minimal overlap, thereby justifying the use of both versions.

**Figure 4:**
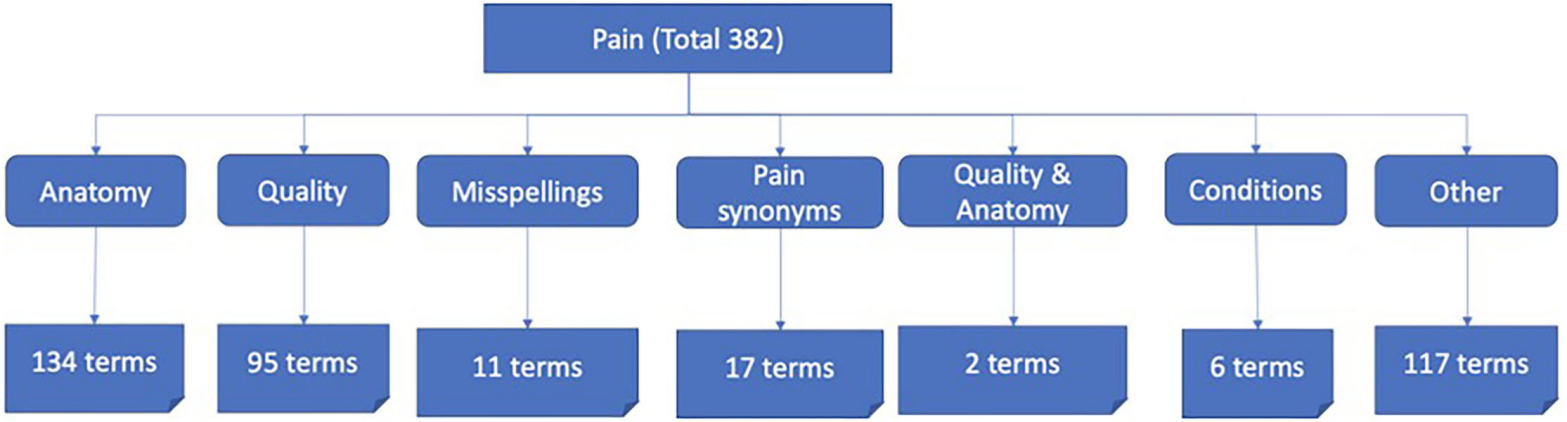
Distribution of terms within pain lexicon.

After post-processing to remove duplicates, punctuations/symbols, and words of less than four characters, the lexicon was validated by two clinicians, leading to a final size of 382 terms (Figure 4).

The final pain lexicon and the code to generate the embedding models is openly available on GitHub^4^ and will also be added to other ontology collections such as BioPortal^5^.

Some patterns were identified within the lexicon which enabled generation of a shorter list of pain terms which captured all the other terms within the patterns, such as the word “pain” capturing “chest pain,” “burning pain,” and ache capturing “headache,” “belly ache,” etc. For example, terms such as “chest pain,” “head discomfort,” “aching muscles,” follow a pattern of <anatomy> followed by <pain term> or vice versa; terms like “burning pain” and “chronic pain” follow a pattern of <quality term> <pain term>, and some are a combination of quality and anatomy such as “chronic back pain” which follows a pattern of <quality term> <anatomy term> <pain term>.

A frequency count of some other common pain related terms [using wildcard character (%) to capture any words containing these terms] was conducted on a cohort of SMI patients within the CRIS EHR documents. Top 13 terms are listed out in Table 8.

**Table 8:** Top 13 common pain-related terms within a cohort of patients (*n* = 57,008) in the CRIS database.

**Keyword**	**Percentage (Over entire cohort) (%)**
%ache%	54
%pain%	36
%burn%	7
%sore%	3
%algia%	< 1
%spasm%	< 1
%dynia%	< 1
%algesia	< 1
colic%	< 1
hurt%	< 1
sciatic%	< 1
tender%	< 1
cramp%	< 1

### Validation of the Lexicon

Two forms of validation were carried out on the lexicon—validation by two clinicians, and validation against an existing ontology of pain terms.

Upon validation by the clinicians, 11 new terms were added to the lexicon and 39 terms were removed from the lexicon. The reasons for removal of words were when they were too ambiguous and non-specific (such as fatigue and complaints), and words that did not indicate pain per se (such as itchiness, nausea, paresthesia, tightness). Some examples of terms that were removed are algophobia, bloating, fatigue, and nausea. Terms added were acronyms (such as LBP for lower back pain), pain education, antalgic gait.

The Experimental Factor Ontology (21) contains a pain sub-section consisting of 26 pain related terms. Upon comparison with our lexicon, it was found that 18 (69%) of the terms within the Experimental Factor Ontology matched. Amongst the ones that did not match, most were words with three tokens, which would have been excluded from our lexicon. The remaining unmatched terms were limb pain, renal colic, pain in abdomen, multisite chronic pain, lower limb pain, episodic abdominal cramps, chronic widespread pain, and abdominal cramps. However, all the pain-related terms (such as cramp, colic, ache, etc.) did match with our lexicon, ensuring the synonyms of pain were indeed all captured.

Medical Subject Headings headings consist of 63 pain-related MeSH terms and 941 pain-related entry terms. Upon comparison with our lexicon, an overlap of 56 terms (89%) was found with the MeSH terms and 649 terms (69%) with the entry terms. The MeSH terms that did not match (11% i.e., seven terms) were not explicitly related to pain, and included terms such as agnosia [a sensory disorder where a person is unable to process sensory information (49)], pramoxine (a topical anesthetic), and generic somatosensory disorders. The entry terms that did not match (33% i.e., 307 terms) consisted of drug names (2% of total terms, 5% of non-matched terms) such as Pramocaine and Balsabit, disorders and syndromes (20% of total terms, 62% of non-matched terms) such as visual disorientation syndrome and Patellofemoral syndrome, generic terms (10% of total terms, 31% of non-matched terms) such as physical suffering, and tests (1% of total terms, 3% of non-matched terms) such as Formalin test. The pain specific terms within this list were mainly pain (50% of total terms), -algia (8%), ache (7%), -dynia (1%), and -algesia (1%). Two new pain terms discovered within this list were “catch” and “twinge” which might reference pain in the right context but could also lead to false positives when used in NLP tasks to identify mentions of pain.

## Discussion and Conclusion

When looking at how pain was mentioned in the different text sources, most mentions fell into similar themes i.e., quality of pain, feelings/experiences associated with the pain, medications, and other measures for pain relief, and mentions of different body parts associated with the pain. The mentions within MIMIC-III were geared more toward pain relief, which is likely due to the data being from critical care units. In contrast, CRIS covered the feelings and experiences associated with pain. It was hard to get a good sense of the Twitter mentions owing to the short length of strings, while Reddit was a lot more detailed around patient experiences, and pain relief remedies.

The information gained from this exploration helped decide the sources for the development of the pain lexicon. Embedding models built using MIMIC-II/III and CRIS databases were used. The final lexicon consisted of 382 pain-related terms. Embedding models built using Twitter (50) and Reddit (32) data were excluded from inclusion into the final lexicon due to the terms not being very relevant to the term “pain.” They generated terms such as brain, anger, patience, and habit with Twitter, and words such as apartment, principal, and goal by Reddit. The Venn diagrams demonstrated the benefits of including different sources as each of these sources provided unique terms thereby enriching the lexicon for pain. CRIS and MIMIC contributed 68 unique terms that are used in “real-life” settings to the final lexicon. These mostly consisted of commonly used words like soreness, pain, aches. Many of these mentions are potentially based on what patients have said, which could also explain why they are a smaller number of terms. The literature and ontologies have a greater variety of words, as they either use more technical terms, or enumerate every term and concept associated with pain. Apart from helping build the lexicon, this exploration will also help further planning for development of NLP applications and deciding on what attributes around pain might be of interest for general and clinical research purposes.

The final lexicon has been validated by two clinicians, compared to an existing Experimental Factor Ontology which consisted of 26 pain-related terms, and MeSH headings and terms (63 pain-related heading terms and 491 pain-related entry terms). The majority of the pain-related terms from both these sources matched those included within the lexicon. The terms that did not match were names of disorders/syndromes that may have pain as a symptom, and other more generic words that could lead to false positives if used in downstream NLP tasks.

This study has several limitations. Most importantly, only a small sample of documents was reviewed for the exploration step. Reviewing a larger sample might have been more representative of the text sources and might have revealed deeper insights. The process of exploration of pain concepts within different sources also highlighted the ambiguous nature of a word like pain, and the different contexts that could contain these mentions (metaphorical or clinical mentions). These factors are important to bear in mind when attempting to use such ambiguous terms in NLP tasks as they could lead to false positive results.

The final lexicon, and the code used to generate the embedding models, have been made openly available. This final lexicon will be used in downstream tasks such as building an NLP application to extract mentions of pain from clinical notes which will in turn help answer important research questions around pain and mental health. The approach followed for the development of this lexicon could be replicated for other clinical terms. Future work includes patient engagement in order to elicit feedback on the terms that have been included in the lexicon. In addition to this, the lexicon will be formalized for submission to portals, such as BioPortal, for wider use by the community.

## Data Availability Statement

The datasets presented in this study can be found in online repositories. The names of the repository/repositories and accession number(s) can be found below: https://github.com/jayachaturvedi/pain_lexicon.

## Author Contributions

The idea was conceived by JC, AR, and SV. JC conducted the data analysis and drafted the manuscript. AR and SV provided guidance in the design and interpretation of results. AM provided scripts and guidance on building some of the embedding models. All authors commented on drafts of the manuscript and approved the final version.

## Funding

AR was funded by Health Data Research UK, an initiative funded by UK Research and Innovation, Department of Health and Social Care (England) and the devolved administrations, and leading medical research charities. AR receives salary support from the National Institute for Health Research (NIHR) Biomedical Research Center at South London and Maudsley NHS Foundation Trust and King's College London. JC was supported by the KCL funded Center for Doctoral Training (CDT) in Data-Driven Health. AM was funded by Takeda California, Inc. This paper represents independent research part funded by the National Institute for Health Research (NIHR) Biomedical Research Center at South London and Maudsley NHS Foundation Trust and King's College London. The funders had no role in study design, data collection and analysis, decision to publish, or preparation of the manuscript. This study received funding from Health Data Research UK, KCL funded CDT in Data-Driven Health, and Takeda California, Inc. The funders were not involved in the study design, collection, analysis, interpretation of data, the writing of this article or the decision to submit it for publication.

## Author Disclaimer

The views expressed are those of the authors and not necessarily those of the NHS, the NIHR or the Department of Health and Social Care.

## Conflict of Interest

The authors declare that the research was conducted in the absence of any commercial or financial relationships that could be construed as a potential conflict of interest.

## Publisher's Note

All claims expressed in this article are solely those of the authors and do not necessarily represent those of their affiliated organizations, or those of the publisher, the editors and the reviewers. Any product that may be evaluated in this article, or claim that may be made by its manufacturer, is not guaranteed or endorsed by the publisher.

## Footnotes

^1^ https://www.reddit.com

^2^ https://twitter.com/?lang=en

^3^ https://www.ncbi.nlm.nih.gov/mesh/?term=pain

^4^ https://github.com/jayachaturvedi/pain_lexicon

^5^ https://bioportal.bioontology.org/
